# Diagnostic Performance of the Pluslife MiniDock MTB and Molbio MTB Ultima Assays to Detect Tuberculosis From Tongue and Sputum Swabs Among Outpatients and in Active Case Finding in Cameroon

**DOI:** 10.1093/cid/ciaf709

**Published:** 2025-12-23

**Authors:** Cyrille Mbuli, Jean Bosco Taya-Fokou, Rita Nsamenang, Nestor Biatu, Gildas Nguimfack, Ngha Ndze Mbuh, Joceline Konso, Zourriyah Adamou Mana, Arthur Rausmis Nankouo-Njouonang, Magdalene Tiamuh, Denis Nsame, Norah Nyah Ndi, Irene Adeline Goupeyou Wandji, Mercy Fundoh, Maurice Ganava, Ousmanou Bello, Paul Meoto, Adeline Fitame, Comfort Vuchas, Teyim Pride Mbuh, Valerie Flore Donkeng-Donfack, Tushar Garg, Jacob Creswell, Melissa Sander, the RAPID TB Consortium, Nina Lubeka, Armand Koudjou, Guy Zero Molesa, Joel Wepngong Tabah, Ngowo Eyambe Lydia, Christabelle Ewane, Nguifu Kelly Ngwafung, Angela Neh, Ambo Noeline Niba, Kolla Magang Nelly Celestine, Bechesi Carine, Boum Delphine Agnes Gaetan, Ngu Amanda Mambo, Yinkfu Marcel Ndamnsah, Chifu Nadege Bonkar, Asonganyi Etiendem, Munsi Valerie Nformi, Hella Adzoyo Reine, Elimbi Neline Yameni, Ahmadou Djenabou, Maiyanpa Moksala Josephine, Diana Gladys Kolieghu, Nchu-Nfor Evangeline, Iya A Nyam Aicha, Laban Rhoda Bongshe, Ngapgue Sobdong Dominique Josiane, Tsimene Tamessuing Sandrine, Gingir Beatrice Ndemnwi, Amaya Alhadji Wakar, Souk-ino Wappou Ferdinand, Guidzavai Koliye Paul, Naindouba Beninga Herve Steve, Eban Odi Luisa Nang, Tchindo Dargeo, Edzengte Pascal Steve Landry, Elisée Avaikdepainani, Njikam Daouda Momgbet, Fonyuy Gilford, Mbah Romarick, Baiguerel Erika Myriam

**Affiliations:** Center for Health Promotion and Research, Bamenda, Cameroon; Center for Health Promotion and Research, Bamenda, Cameroon; Center for Health Promotion and Research, Bamenda, Cameroon; Center for Health Promotion and Research, Bamenda, Cameroon; Center for Health Promotion and Research, Bamenda, Cameroon; Center for Health Promotion and Research, Bamenda, Cameroon; Center for Health Promotion and Research, Bamenda, Cameroon; Center for Health Promotion and Research, Bamenda, Cameroon; Center for Health Promotion and Research, Bamenda, Cameroon; Center for Health Promotion and Research, Bamenda, Cameroon; Bamenda Regional Hospital, Bamenda, Cameroon; Cameroon Baptist Convention Health Services, Bamenda, Cameroon; National TB Program—Littoral Region, Douala, Cameroon; National TB Program—Northwest Region, Bamenda, Cameroon; National TB Program—Far North Region, Maroua, Cameroon; National TB Program—North Region, Garoua, Cameroon; National TB Program—Southwest Region, Buea, Cameroon; National TB Program—West Region, Bafoussam, Cameroon; Center for Health Promotion and Research, Bamenda, Cameroon; Tuberculosis Reference Laboratory Douala, Douala, Cameroon; Centre Pasteur du Cameroun, Yaoundé, Center, Cameroon; Stop TB Partnership, Geneva, Switzerland; Stop TB Partnership, Geneva, Switzerland; Center for Health Promotion and Research, Bamenda, Cameroon

**Keywords:** tuberculosis, diagnostic, PlusLife, Molbio

## Abstract

**Background:**

Cost and infrastructure requirements limit access to current rapid molecular diagnostic testing for tuberculosis (TB). Recent developments in novel, swab-based assays that can be used closer to the point of care offer the potential to change the TB diagnostic landscape.

**Methods:**

We assessed the diagnostic accuracy of 2 tests, Molbio Truenat MTB Ultima (MTB Ultima) and Pluslife MiniDock MTB Test (MiniDock MTB), among consecutively enrolled individuals aged 15 years and older, against the reference standard of culture and comparators of microscopy and Xpert MTB/RIF Ultra. Two tongue swabs, either self-collected or healthcare worker collected, and 2 sputum specimens were requested from each participant; MiniDock MTB was tested the same day and MTB Ultima was tested after storage.

**Results:**

From February to June 2025, 1097 participants were enrolled in communities (382) and at health facilities (715). Sensitivities of sputum and tongue swabs on MiniDock MTB among 132 people with culture-positive TB were 86% (95% confidence interval [CI], 79–91) and 76% (95% CI, 68–82), respectively, and 67% (95% CI, 51–79) and 44% (29–59) among those with smear-negative TB. Sensitivities of sputum and tongue swabs on MTB Ultima were 84% (97/116; 95% CI, 76–89) and 74% (67/91; 95% CI, 64–82), respectively, and 58% (95% CI, 41–74) and 33% (95% CI, 18–53) among those with smear-negative TB.

**Conclusions:**

In this population, the performance of both MiniDock MTB and MTB Ultima on tongue and sputum swabs was similar to target product profile thresholds for near point-of-care TB tests. Further studies to evaluate performance in diverse populations and settings are needed.

## Teaser

Our diagnostic accuracy study of new swab-based MiniDock MTB and MTB Ultima assays in Cameroon demonstrated accuracies similar to the tuberculosis detection target product profiles overall for near point-of-care tongue and sputum swabs.

Tuberculosis (TB) kills more people globally (1.25 million in 2023) than any other infectious disease, more than human immunodeficiency virus (HIV) and malaria combined [1]. This is largely because of the roughly 2.5 million people with incident TB missed by health systems each year alongside millions more missed with prevalent disease [1].

Sputum microscopy had been the cornerstone of TB diagnosis for decades, but has limited sensitivity, especially among people with HIV and children, and is highly dependent on human performance [2]. In 2010, the World Health Organization (WHO) recommended the use of Cepheid's (Sunnyvale, USA) GeneXpert system and Xpert MTB/RIF (Xpert) cartridge-based automated nucleic acid amplification test (NAAT), as a diagnostic with much higher sensitivity to detect TB [3]. In 2016, a manual NAAT platform, the Loopamp MTBC assay (TB LAMP, Eiken, Japan), was introduced as a lower cost alternative for the detection of TB [4]. The next-generation Xpert MTB/RIF Ultra (Ultra) further improved sensitivity compared to the Xpert [5], and together with the Molbio Diagnostics (Goa, India) Truenat platform [6,7], these NAATs have become the global standard for the detection of TB and rifampin resistance [8].

Unfortunately, in 2023, fewer than half of people with TB received a rapid molecular assay as their initial diagnostic test [1,9]. This shortcoming can largely be attributed to cost and complexity. Even at current prices of just under USD 8, NAATs have expensive modular components and maintenance and service add to real per-test costs [10, 11]. Additionally, the infrastructure needed to use the platforms has generally relegated current low-complexity NAAT devices to district level care, whereas many patient pathway analyses have shown that people with TB first seek care at lower levels in the healthcare system [12, 13].

Novel, simplified diagnostic platforms to test oral swabs and swabbed sputum have the potential to be placed at lower levels of care to increase access to testing [14]. One such assay is Molbio MTB Ultima (Goa, India) with the new Truelyse device that replaces the Trueprep extraction device with a 90-second sonication lysis step, followed by real-time polymerase chain reaction on the Truelab instrument (∼35 minutes). The other is MiniDock MTB (Guangzhou Pluslife Biotech, China) with an initial 5-minute lysis step (Thermolyse) followed by the MiniDock instrument for a 25-minute (or less for some positive specimens) combined amplification and detection step [15]. Steadman et al reported on the performance of these 2 platforms for tongue and sputum swabs in outpatients in India, Uganda, and Vietnam [16]. In the populations tested, including 95% of people with cough >2 weeks, the sensitivity of sputum swabs to detect TB on both platforms was similar to Ultra on sputum, and the sensitivity of tongue swabs was significantly better than sputum smear microscopy, all with high specificities.

Additional evaluations of the performance of these new assays to detect TB are needed, in different populations and settings, including in the community. We report on a diagnostic accuracy study of both platforms among individuals enrolled in facility and community settings in Cameroon.

## METHODS

### Study Design and Participants

This was a prospective, multisite, diagnostic accuracy study of the performance of the MiniDock MTB and MTB Ultima assays conducted in Cameroon, at 5 health facilities and as part of active case finding in community health camps (Supplementary Table 1).

At health facilities, consecutive outpatients aged 15 years and older with symptoms (current cough, fever, night sweats, and/or weight loss) and/or clinical risk factors (diabetes, current smoker) for TB were invited to participate. At community-based active case finding (ACF) events, organized in collaboration with community leaders in rural areas of the North and Far North regions, people aged 15 years and older were screened using chest X-ray with artificial intelligence (qXR V4, Qure.ai, Mumbai, India), and those with a score of ≥0.30 were invited to participate. Individuals who were on TB treatment within the previous 6 months were excluded.

### Procedures

After providing written informed consent, participant demographic information and medical history were recorded. Two tongue swabs and 2 sputum specimens were requested from each participant (Supplementary Figure 1).

#### Specimen Collection

Participants were shown a short video on how to self-collect tongue swabs, including swabbing the dorsum while rotating the swab for at least 30 seconds, in line with the consensus protocol [17]. Then the participant was asked if they would self-collect a specimen under supervision; for participants who elected not to self-collect, a healthcare worker collected the tongue swabs. Swabs were collected after ensuring that the participant had consumed nothing by mouth within 30 minutes, with nylon, flocked swabs (Copan FLOQSwabs 502CS01 or 520CS01) and stored dry in sterile tubes. Two swabs were collected successively; the testing sequence was randomized (Supplemental methods) [18].

Following tongue swab collection, participants were instructed on how to produce 2 sputum specimens with a volume of ≥2 mL each, collected at least 30 minutes apart. Participants were shown an adapted 2-minute video on how to produce sputum for TB testing in 1 of 4 languages (English, French, Fulfulde, Pidgin) [19]; in some cases, people who had difficulty producing sputum were offered the Lung Flute ECO (Acoustic Innovations, Japan) with video coaching on how to use this device to assist sputum collection [20].

From each sputum specimen, a smear was prepared for microscopy, and 2 swabs were prepared by swirling the swab in sputum 10 times over 10–15 seconds, then stored in sterile dry tubes until testing.

Swabs for MiniDock MTB were stored at room temperature and tested within 4 hours or stored at 2–8 °C and tested within 24 hours; swabs for MTB Ultima were transferred to the reference lab and stored at −20 °C for up to 5 months before testing. The second sputum specimen was stored at 2–8 °C and transported to the reference laboratory for culture.

#### Index Tests

MiniDock MTB tests were performed onsite during 46 ACF events and in 1 of 2 laboratories (Supplementary Table 1). Tests were performed following manufacturer's instructions. The MiniDock MTB provides results of positive, negative, or invalid (Supplementary Table 2).

Ultima MTB tests were performed at the reference lab on stored specimens, which were first thawed to room temperature and then tested following the manufacturer's instructions, with results from the TrueLab as MTB detected (high, medium, low, or very low), MTB not detected, or invalid.

Invalid tests were repeated once, with the repeat result reported. Index tests were performed by technicians without knowledge of any other TB test results or clinical information.

### Reference Standard and Comparator Tests

The reference standard for this study was TB culture, and comparator tests were smear microscopy and Ultra, as recommended for evaluations of sputum-based tests to detect TB [21]. At the reference laboratory, sputum was processed for culture using the N-acetyl L-cysteine and sodium hydroxide (NALC-NaOH) method and inoculated onto liquid (mycobacterial growth indicator tubes [MGIT], BD Diagnostic Systems, USA) and solid media (Supplemental methods) [22]. Smear microscopy was performed by Ziehl-Neelsen or fluorescence microscopy. The remainder of the first sputum was used for Ultra testing, following the manufacturer's instructions. The Xpert MTB/RIF Ultra assay produces results of invalid, error, MTB not detected, or MTB detected (high, medium, low, very low, or trace). Technicians performing testing were blinded to index test results and clinical information.

### Data Analysis

The sample size was calculated as a minimum of 112 people with culture-positive TB, using a sensitivity target of 75% (based on the target product profile [TPP] for near-point-of-care, nonsputum specimens) [23] and a 95% confidence interval (CI) of ±8%.

**Figure 1.**
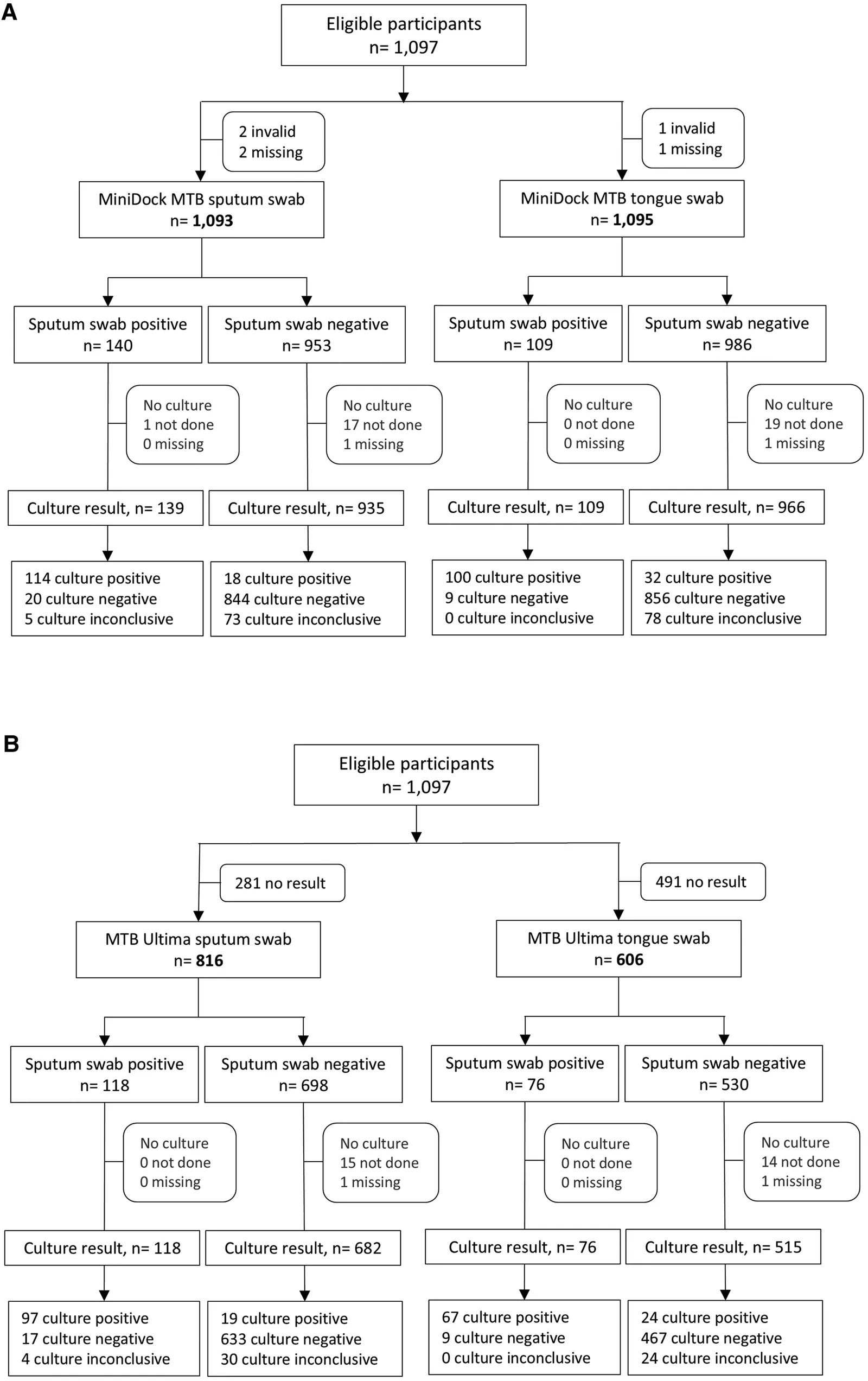
Participant flow (*A*) MiniDock MTB testing (*B*) MTB Ultima testing.

For the diagnostic accuracy analysis, a microbiological reference standard based on culture from 1 sputum specimen was used, including 1 automated liquid culture and 1 solid culture (Figure 1). Participants for whom at least 1 culture was positive for TB were defined as having culture-positive TB; among these, anyone with at least 1 smear positive for TB was defined as smear positive. Participants for whom MGIT or both cultures were negative for TB were defined as culture negative for TB; participants for whom culture results were contaminated or missing were excluded from the analysis. For the comparator, any Ultra result with MTB detected was considered positive. A diagnostic agreement analysis of the index tests was performed against the Ultra comparator.

Participants were characterized using simple descriptive statistics. To evaluate the accuracy of the MiniDock MTB and MTB Ultima assays against the culture reference standard, we calculated the sensitivity and specificity point estimates with 95% CI) using the Wilson score method; the same approach was used to obtain positive and negative percent agreement estimates against the Ultra comparator. The proportion of nonactionable results for the MiniDock MTB index test was reported; because testing on the Ultima MTB was not conducted on fresh specimens, only tests with valid results were included in the analysis and the rate of nonactionable results was not calculated for this assay. The Standards for Reporting of Diagnostic Accuracy recommendations were followed for reporting these data (Supplementary Table 3) [24]. Analyses were performed in R version 4.5.1.

This study was approved by the National Ethics Committee for Research on Human Health and by the Institutional Review Board of the Cameroon Baptist Convention Health Board. This study was registered at PACTR (202502473140037). Study data were collected and managed using (Research Electronic Data Capture) REDCap tools.

## RESULTS

### Participants

From February to June 2025, 1097 participants were enrolled, with index and reference standard results as shown in Figure 1. Of these, 44% were female, the median age was 49 years, 19% were people known to have HIV, and 132 (12%) had culture-positive TB (Table 1). Ultra grade distribution differed by setting, with 47% of those in ACF having grades of very low or trace, as compared to 17% of outpatients. Overall, 1095 (99.8%) provided a first sputum specimen, 1077 (98.2%) provided 2 sputum specimens, and 1096 (99.9%) provided 2 tongue swab specimens.

**Table 1.** Participant Demographic and Clinical Characteristics, Overall and by Setting

Characteristic	Overall		Active Case Finding (ACF)		Health Facilities	
Total	1097		382		715	
Age, y (median, IQR)	49	(36–63)	52	(40–67)	47	(34–60)
Female	487	(44)	157	(41)	330	(46)
People with HIV^a^	205	(19)	7	(2)	198	(28)
Chest X-ray, AI score (median, IQR)	…	…	0.86	(0.62–0.96)	…	…
Cough > 2 wks	494	(45)	143	(37)	351	(49)
TB symptoms, any of W4SS	920	(84)	327	(86)	593	(83)
Tongue swab collection method	…	…	…	…	…	…
Supervised self-collection	915	(83)	305	(80)	610	(85)
Healthcare worker collection	181	(16)	77	(20)	104	(15)
Sputum Ultra positive	163	(15)	57	(15)	106	(15)
High	61	(37)	6	(11)	55	(52)
Medium	23	(14)	6	(11)	17	(16)
Low	34	(21)	18	(32)	16	(15)
Very low	19	(12)	15	(26)	4	(4)
Trace	26	(16)	12	(21)	14	(13)
Sputum TB culture positive	132	(12)	32	(8)	100	(14)
Sputum smear-positive^b^	90	(68)	19	(59)	71	(71)
Sputum smear-negative	39	(30)	13	(41)	26	(26)

Abbreviations: HIV, human immunodeficiency virus; IQR, interquartile range; TB, tuberculosis; W4SS, World Health Organization-recommended 4-symptom screen for tuberculosis.

^a^101 with HIV status unknown (all in active case findings).

^b^3 with smear result missing.

### MiniDock MTB Diagnostic Accuracy and Agreement

For the MiniDock MTB, overall sensitivity of sputum and tongue swabs as compared to the culture reference standard were 86% (95% CI, 79–91) and 76% (95% CI, 68–82), respectively, as compared to sputum Ultra of 95% (95% CI, 90–98) (Table 2). Among smear-negative specimens, the sensitivity of sputum swabs was 67% (95% CI, 51–79), of tongue swabs 44% (95% CI, 29–59), as compared to sputum Ultra of 85% (95% CI, 70–93). Among people who did not report cough more than 2 weeks, the sensitivity of sputum swabs on the MiniDock MTB was 66% (95% CI, 47–80) and of tongue swabs was 52% (95% CI, 34–69) (Supplementary Tables 4 and 5).

**Table 2.** Diagnostic Accuracy of Tongue Swabs and Sputum Swabs on MiniDock MTB and Sputum on Xpert MTB/RIF Ultra for Detection of TB, as Compared With the Reference Standard of TB Culture

	N^a^	Sensitivity: All Culture Positive, % (95% CI, N/N)		Sensitivity: Smear Positive^b^, % (95% CI, N/N)		Sensitivity: Smear Negative, % (95% CI, N/N)		Specificity^c^ % (95% CI, N/N)	
Sputum—Xpert MTB/RIF Ultra	998	95%	(90–98)	100%	(96–100)	85%	(70–93)	97%	(96–98)
…	…	126/132		90/90		33/39		842/866	
Sputum swab—MiniDock MTB	996	86%	(79–91)	96%	(89–98)	67%	(51–79)	98%	(96–99)
…	…	114/132		86/90		26/39		844/864	
Tongue swab—MiniDock MTB	997	76%	(68–82)	90%	(82–95)	44%	(29–59)	99	(98–99%)
…	…	100/132		81/90		17/39		856/865	

Abbreviation: TB, tuberculosis.

^a^Number of participants with valid results on culture and index or comparator test.

^b^3 with missing smear results.

^c^2 invalid sputum swab results, 1 invalid tongue swab result.

As compared to sputum on Ultra, positive percent agreement of sputum swabs on the MiniDock MTB was 86% (95% CI, 78–91) at health facilities and 58% (95% CI, 45–70) in community ACF, with poorer agreement in participants with sputum Ultra results of very low and trace (Table 3). Positive percent agreement of tongue swabs was 75% (95% CI, 66–83) at health facilities and 42% (95% CI, 30–55) in the community.

**Table 3.** Diagnostic Agreement of Tongue Swabs and Sputum Swabs on MiniDock MTB With Sputum on Xpert MTB/RIF Ultra, by Result Grade and Setting

	Positive Percent Agreement								
	Health Facilities			Active Case Finding			Negative Percent Agreement		
	% (95% CI)		n/N	% (95% CI)		n/N	% (95% CI)		n/N
**Sputum swabs** (n = 1093)	…	…	…	…	…	…	…	…	…
Overall	86%	(78–91)	91/106	58%	(45–70)	33/57	98.3%	(97–99%)	914/930
High	93%	(83–97)	51/55	100%	(61–100)	6/6	…	…	…
Medium	100%	(82–100)	17/17	100%	(61–100)	6/6	…	…	…
Low	94%	(72–99)	15/16	83%	(61–94)	15/18	…	…	
Very low	75%	(30–95)	3/4	27%	(11–52)	4/15	…	…	…
Trace	36%	(16–61)	5/14	17%	(3–49)	2/12	…	…	…
**Tongue swabs** (n = 1094)	…	…	…	…	…	…	…	…	…
Overall	75%	(66–83)	80/106	42%	(30–55)	24/57	99.5%	(99–100%)	926/931
High	95%	(85–98)	52/55	83%	(44–97)	5/6	…	…	…
Medium	76%	(53–90)	13/17	100%	(61–100)	6/6	…	…	…
Low	75%	(51–90)	12/16	67%	(44–84)	12/18	…	…	…
Very low	50%	(15–85)	2/4	7%	(1–30)	1/15	…	…	…
Trace	7%	(1–31)	1/14	0%	(0–25)	0/12	…	…	…

### MTB Ultima Diagnostic Accuracy and Agreement

Following testing of stored specimens after thawing, 281 sputum swab and 491 tongue swab specimens without valid results were not included in the analyses (Figure 1).

Among 766 MTB Ultima results on sputum swabs, overall sensitivity was 84% (95% CI, 76–89) and sensitivity among smear-negative specimens was 58% (95% CI, 64–91), as compared to 95% (95% CI, 89–98) overall and 81% (95% CI, 64–91) for smear-negative specimens on sputum Ultra (Table 4 and Supplementary Tables 6 and 7).

**Table 4.** Diagnostic Accuracy of Sputum Swabs and Tongue Swabs on MTB Ultima and Sputum on Xpert MTB/RIF Ultra for Detection of TB, as Compared With the Reference Standard of TB Culture

	N^a^	Sensitivity: All Culture Positive, % (95% CI, N/N)		Sensitivity: Smear Positive^b^, % (95% CI, N/N)		Sensitivity: Smear Negative, % (95% CI, N/N)		Specificity % (95% CI, N/N)	
Sputum—Xpert MTB/RIF Ultra	766	95%	(89–98)	100%	(96–100)	81%	(64–91)	97%	(96–98)
…	…	110/116		82/82		25/31		633/650	
Sputum swab—MTB Ultima	766	84%	(76–89)	94%	(87–97)	58%	(41–74)	97%	(96–98)
…	…	97/116		77/82		18/31		633/650	
Sputum—Xpert Ultra	565	96%	(89–98)	100%	(95–100)	83%	(64–93)	98%	(96–99)
…	…	87/91		67/67		20/24		467/476	
Tongue swab—MTB Ultima	565	74%	(64–82)	88%	(78–94)	33%	(18–53)	98%	(96–99)
…	…	67/91		59/67		8/24		467/476	

Abbreviation: TB, tuberculosis.

^a^Number of participants with valid results on culture and index or comparator test. Testing on MTB Ultima was performed on specimens that were transported and stored at −20 °C for up to 5 months prior to testing (rather than testing shortly after collection). The subset of results presented here are for the specimens that had valid results after thawing and testing; this subset of results is not representative of the population tested. Agreement with Ultra is summarized in Supplementary Table 6.

^b^3 with missing smear results.

Among 565 MTB Ultima results on tongue swabs, overall sensitivity was 74% (95% CI, 64–82) and sensitivity among smear-negative specimens was 33% (95% CI, 18–53), as compared to 96% (95% CI, 89–98) overall and 83% (95% CI, 64–93) for smear-negative specimens on sputum Ultra.

As compared to Ultra on sputum, the positive percent agreement of Ultima varied by Ultra grade, from 96% (55/57; 95% CI, 88–99) for sputum swab specimens from participants with Ultra High results to 0% (0/9; 95% CI, 0–30) for tongue swabs from participants with Ultra Trace results (Supplementary Table 8).

### Tongue Swab Collection Self vs Healthcare Worker

On tongue swabs tested on MiniDock TB, sensitivity of TB detection among people who elected supervised self-collection was 75% (84/112; 95% CI, 66–82), similar to the sensitivity of 80% (16/20; 95% CI, 58–92) with healthcare worker collected tongue swabs (Supplementary Table 5). For tongue swabs tested on Ultima, sensitivity for self-collection was 73% (60/82; 95% CI, 63–82), similar to 78% (7/9; 95% CI, 45–94) for healthcare worker collected specimens (Supplementary Table 8).

### MiniDock MTB Test Success Rate, Time to Result, and Operating Temperature

On the MiniDock MTB, the initial test invalid rate for sputum swabs was 1.0% (11/1096 tests) and for tongue swabs was 0.9% (10/1096 tests); on repeat testing, 2/11 and 1/10, respectively, were invalid (Supplementary Table 9).

The MiniDock MTB instrument test time to generate negative results is 25 minutes; for positive results, the average time to result was 13.4 minutes (SD = 4.0 minutes) for sputum swabs and 14.3 minutes (SD = 4.3 minutes) for tongue swabs (Supplementary Table 10).

At ACF events, the MiniDock MTB test was routinely performed at temperatures of >35 °C (Supplementary Table 11).

## DISCUSSION

We report a diagnostic evaluation of MTB Ultima and MiniDock MTB assays, including people recruited in community settings as well as at health facilities. Our results demonstrated performance of both assays to be robust, with swabbed sputum similar to Ultra among individuals with higher semiquantitative results on Ultra, but with poorer performance among individuals with lower semiquantitative results. In this population, MiniDock MTB met the WHO TPP thresholds for both sputum and nonsputum based near point-of-care tests, whereas MTB Ultima was within a percentage point of the targets [23]. These results are similar to another early study in health facilities for both the diagnostic performance of both the assays and the low rates of test failure for MiniDock MTB [16].

The performance of both assays was less sensitive on tongue swabs in general and among individuals with lower semiquantitative results on Ultra. The differences across semiquantitative grades on Ultra may be important based on the population being tested. Although using a small sample, our results suggest that people with TB identified in community settings may have lower levels of TB bacilli and a different semiquantitative results profile than those identified in health facilities [25]. Nevertheless, because most TB programs in high-burden countries still test large numbers of people with microscopy, an improvement in performance compared to microscopy coupled with increased coverage at point of care could greatly enhance coverage.

Overall, staff running the tests reported both platforms were easy to use with minimal training, and workflows were straightforward enough to be done at a health facility with basic staffing and electricity. The Pluslife platform was also used directly in the field at ambient temperatures between 35–40 °C (Supplementary Table 11), with a solar power supply for the Thermolyse.

The Pluslife MiniDock TB assay has the advantage of running on an extremely low-cost instrument (less than $200 each for the MiniDock MTB and Thermolyse tools), with tests that are already much lower cost than currently available NAATs (MiniDock TB <$4 vs TB LAMP $6 when optimally batched, Truenat $8, Ultra $8 [16, 26]) and may be even less expensive when supplied at higher volumes. The platform is >20 times less expensive than either GeneXpert and Truelab instruments and >8 times less expensive than the TB LAMP instrument. However, a major disadvantage of MiniDock MTB platform as compared to the existing automated NAAT platforms is that no rifampin resistance testing is possible. This disadvantage is shared by the TB LAMP platform and has contributed to slow uptake of this technology, in part because of the need to provide subsequent rifampin-resistance testing on a separate platform [4].

The MTB Ultima assay is performed with lysis on the new Truelyse instrument followed by TB detection on the Truelab instrument. The Truelab instrument is already part of the platform used for testing the Truenat MTB Plus assay, which has been recommended by the WHO since 2020, with the Truenat MTB-RIF assay for detection of rifampin resistance. The Truelab is a real-time polymerase chain reaction instrument available for $10 000 and higher [26]. The platform is portable and battery-powered, with demonstrated good performance for TB and rifampin resistance detection in peripheral and community settings [8]. The MTB Ultima assay has the advantage that rifampin resistance testing can be performed with the same Truelab instrument, but Truelab's high cost (at least 5 times the WHO TPP capital cost target of $2000) [23] may represent a significant barrier for scale-up and decentralization.

The ability of both platforms to test oral swabs presents both opportunities and implementation questions. Diagnostic performance of tongue swabs collected by participants under supervision and by healthcare workers was similar in this study (Supplementary Tables 5 and 8). Previous studies have documented large losses in the diagnostic cascade related to sputum collection and testing [27–29] that an easy-to-use tongue swab test could potentially address to increase diagnostic yield [14, 30]. Another early study of swab-based testing platforms reported similar diagnostic yield between tongue swab and swabbed sputum across 4 countries [31], although only 85% of participants overall provided sputum. In our study, 99% of individuals provided sputum, including in community settings, aided by video coaching and in some cases the use of Lung Flute ECO; similar high sputum collection rates have been documented in other work [32, 33]. Performance of tongue swabs was lower than swabbed sputum, as expected [34, 35]; in addition, among the relatively small number of people with HIV included in this study, the sensitivity of tongue swabs to detect TB was lower than in people without HIV (Supplementary Tables 5 and 7). During programmatic implementation, highlighting the importance of obtaining sputum when possible to improve detection will be critical.

Another approach to facilitate greater access to rapid diagnostic testing is pooled testing. Several studies of pooled testing on Ultra have shown per person testing costs reduced by 30% to 60% with minimal loss in performance in diverse populations [36–38]. More evidence is needed to guide implementers about when to use high-sensitivity tests (eg, pooled or individual Ultra) versus easier to implement tests (eg, MiniDock MTB) to improve both access to testing and detection of TB in different populations, including cost considerations.

Our study has several limitations. We performed culture on 1 sputum per participant rather than on 2 specimens as preferred. In addition, culture was conducted on different a specimen than index testing [21]. MTB Ultima was not tested on fresh samples, but instead after storage. As the test is designed for use on fresh specimens, sample degradation leading to invalid results, for example due to lack of detection of the internal control, would not be expected to impact programmatic implementation. Although we included participants recruited in the community and people with HIV, future work should include larger sample sizes and other populations like children.

## CONCLUSION

MTB Ultima and MiniDock MTB swab testing platforms bring the potential for TB diagnosis nearer to point of care. The platforms are robust, have easy-to-use workflows, and have clear performance improvements over smear microscopy. In certain populations, such as people reporting cough more than 2 weeks, these tests have similar performance to existing low-complexity NAAT platforms, and the MiniDock MTB has a much lower test and equipment cost. Future studies are needed to investigate performance in different populations, especially at lower levels of care, as well as how best to integrate swab testing in laboratory networks, how swab testing is used and received by both people with presumptive TB and health care providers, and cost effectiveness.

## Supplementary Material

ciaf709_Supplementary_Data

## Supplementary Data

Supplementary materials are available at *Clinical Infectious Diseases* online. Consisting of data provided by the authors to benefit the reader, the posted materials are not copyedited and are the sole responsibility of the authors, so questions or comments should be addressed to the corresponding author.
