# Supplementary material for: Diagnostic Performance of the Pluslife MiniDock MTB and Molbio MTB Ultima Assays to Detect Tuberculosis From Tongue and Sputum Swabs Among Outpatients and in Active Case Finding in Cameroon
Source: Clin Infect Dis. 2025 Dec 23;83(2):e370–8. doi: 10.1093/cid/ciaf709 (PMC13537157; doi:10.1093/cid/ciaf709)
Supplement: Supplementary file 1 [file ciaf709_supplementary_data.zip › Supplementary_materials_final.pdf]

Diagnostic performance of the MiniDock MTB and MTB Ultima assays to detect tuberculosis from tongue and sputum swabs among outpatients and in active case finding in Cameroon

Supplementary materials

Table of Contents

1. Supplementary methods 2
   a. Randomization of tongue swab testing order 2
   b. TB culture testing 2
2. Supplemental Figure 3
   Figure S1. Specimen and testing flow 3
3. Supplemental Tables 4
   Table S1. Testing locations for index, comparator, and reference tests, by enrollment site 4
   Table S2. Details of index test reagents used in evaluation 5
   Table S3. STARD checklist 6
   Table S4. Diagnostic accuracy of sputum swabs on the MiniDock MTB assay for detection of TB, as compared with the reference standard of TB culture, by sub-group 8
   Table S5. Diagnostic accuracy of tongue swabs on the MiniDock MTB assay for detection of TB, as compared with the reference standard of TB culture, by sub-group 9
   Table S6. Diagnostic agreement of sputum and tongue swabs on the MTB Ultima assay for detection of TB, as compared with sputum on the Xpert MTB/RIF Ultra assay, by result grade 11
   Table S7. Diagnostic accuracy of sputum swabs on the MTB Ultima assay for detection of TB, as compared with the reference standard of TB culture, by sub-group 10
   Table S8. Diagnostic accuracy of tongue swabs on the MTB Ultima assay for detection of TB, as compared with the reference standard of TB culture, by sub-group 10
   Table S9. Test success rate, MiniDock MTB, by swab type 12
   Table S10. Time to result of MTB positive sputum and tongue swabs on the MiniDock MTB instrument, overall and by Ultra sputum semi-quantitative grade 12
   Table S11. Ambient temperature measured at the time of swab testing with the MiniDock MTB device, by setting and test result 13

1. Supplementary methods

   a. Randomization of tongue swab testing order

      Sequences were assigned in randomly permuted blocks of 50, stratified by site, by a person not involved in the interventions, using a random sequence generator software and sealed in sequentially numbered opaque envelopes. At the testing lab, the technician opened the envelope with the appropriate participant number and placed labels on the storage tubes containing each tongue swab with the name of the index test to be performed based on tongue swab collection order (e.g. specimen # 1-PlusLife/ specimen #2-Ultima or specimen # 1-Ultima/ specimen #2-PlusLife).

   b. TB culture testing

      For the microbiological reference standard (MRS), individuals were considered positive for TB if any culture result was positive. At the reference laboratory, sputum specimens of ≥2 mL were initially homogenized by vortexing the specimens with 3mm sterile glass beads for 20 min. Then, the homogenized sputum was aliquoted for culture processing (2 mL) and any excess was stored in 0.5mL aliquots for additional testing as needed. Sputum was processed using the N-acetyl-L-cysteine–NaOH (NALC-NaOH) method for TB culture.[1] After centrifugation, the pellet was resuspended in the same volume as the starting sputum (2 mL) of phosphate-buffered saline (PBS) buffer; then, 0.5 mL was inoculated on mycobacterial growth indicator tubes (MGIT; BD Diagnostic Systems, Sparks, MD, USA) using the Bactec MGIT 960 system, and 0.2 mL was inoculated on Lowenstein-Jensen media. Cultures positive for acid- fast bacilli were tested for *M. tuberculosis* complex by MPT64 antigen detection (Standard Diagnostics, Republic of Korea). Isolates that were AFB positive and negative by MPT64 antigen testing were tested for mycobacterial identification using the GenoType Mycobacterium CM and AS line probe assays (Hain Lifescience, Germany) according to the manufacturer's instructions.

1. Kent PT, Kubica GP. Public Health Mycobacteriology: a guide for the level III laboratory. Atlanta, Ga.: U.S. Department of Health and Human Services, Public Health Service, Centers for Disease Control, 1985.

2. Supplemental Figure

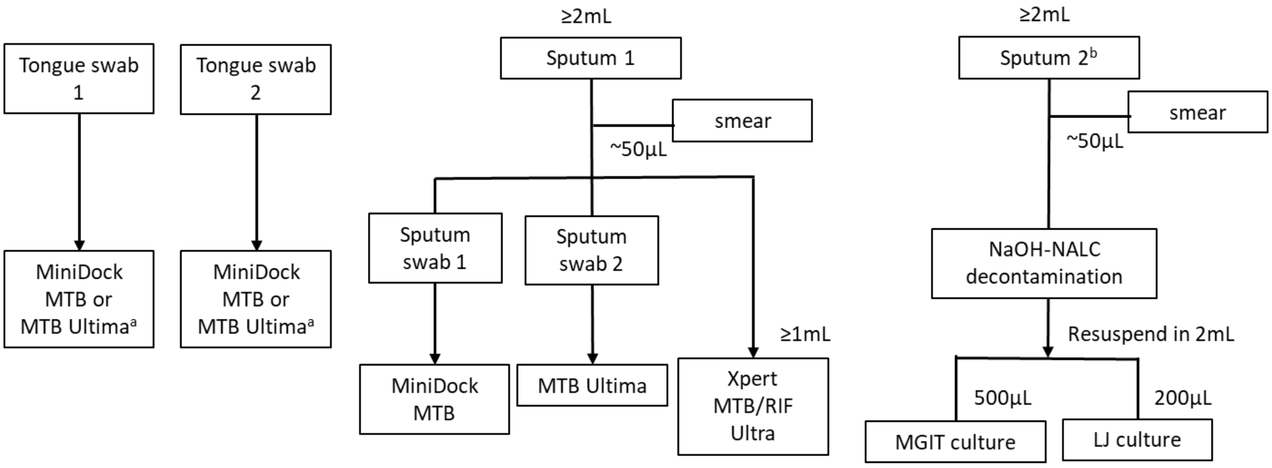

Figure S1. Specimen and testing flow

[a] Tongue swabs were randomized after collection to either #1-MiniDock / #2-Ultima *or* #1-Ultima / #2-MiniDock
[b] 2mL of sputum was processed for culture; specimens with >2mL were homogenized and excess was aliquoted and stored; at health facilities, two additional tongue swabs and a 3rd sputum specimen were collected on Day 2 when possible; stored sputum and pellets were used for additional testing in case of contaminated or discordant results.

Participants were shown an adapted 2-minute video on how to produce sputum for TB testing in one of four languages (English, French, Fulfulde, Pidgin)[2]; in some cases, people who had difficulty to produce sputum were offered the Lung Flute ECO (Acoustic Innovations, Japan) with video coaching on how to use this device to assist sputum collection[3].

2. IRD Global. "Good Sputum, Better Diagnosis" - (English) Instructional video for sputum submission [Internet]. 2013. Available at: https://www.youtube.com/watch?v=92dT_1kbbek. Accessed 3 November 2025.
3. Mbuli C, Vuchas C, Konso J, et al. Use of the Lung Flute ECO assists in sputum collection for tuberculosis testing: A randomized crossover trial. ERJ Open Res **2024**; :00902–02023. Available at: https://doi.org/10.1183/23120541.00902-2023.

3. Supplemental Tables

Table S1. Testing locations for index, comparator, and reference tests, by enrollment site

Enrollment site	Affiliated lab(s) for index and comparator testing	MiniDock MTB test location	MTB Ultima test location[2]	Smear microscopy[3] test location	Ultra test location	TB culture test location[4]
**Health facility 1**	Reference Lab 1	Ref Lab 1	Ref Lab 1	Ref Lab 1	Ref Lab 1	Ref Lab 1
**Health facility 2**	Xpert Lab 1	XP Lab 1	Ref Lab 1	XP Lab 1	XP Lab 1	Ref Lab 1
**Health facility 3**	Xpert Lab 1	XP Lab 1	Ref Lab 1	XP Lab 1	XP Lab 1	Ref Lab 1
**Health facility 4**	Microscopy Lab 1, Ref Lab 1	Ref Lab 1	Ref Lab 1	Mic Lab 1	Ref Lab 1	Ref Lab 1
**Health facility 5**	Ref Lab 1	Ref Lab 1	Ref Lab 1	Ref Lab 1	Ref Lab 1	Ref Lab 1
**Community events[1]**	Xpert Lab 2 or 3	Community event[1]	Ref Lab 1	Ref Lab 1	XP Lab 2 or 3	Ref Lab 1

[1] Community-based active case finding (ACF) events were organized in collaboration with community leaders at community gathering spots, including health posts, markets, schools, crossroads, or at the compounds of traditional leaders in rural areas of the North and Far North regions of the country. Depending on the event location, MiniDock MTB testing was performed onsite either in a temporary lab set up in a building, under a tree, or in the customized LandCruiser that was also used for transporting the team and equipment to the site.

[2] For all MTB Ultima testing, tongue and sputum samples were collected at the various enrollment sites, then transported and stored at the reference lab prior to testing when reagents were available

[3] Ziehl-Neelsen microscopy was performed at Microscopy Lab 1 and Xpert Lab 1; auramine fluorescence microscopy was performed at Ref Lab 1

[4]TB culture was performed at the Tuberculosis Reference Laboratory Bamenda, which is accredited in accordance with the recognized International Standard ISO 15189:2022 (SANAS Accredited Medical Laboratory, no. M0593).

Table S2. Details of index test reagents used in evaluation

**PlusLife MiniDock MTB**		
Ref	RM1012202-10	RM1012202-10
Lot	20240901	20241202
Mfg Date	18/09/2024	12/12/2024
Exp Date	17/10/2025	11/01/2026
**TrueLyse Buffer***		
Ref	950030050	
Lot	02TTLB	
Mfg Date	2025-02	
Exp Date	2027-01	
**Truenat MTB Ultima***		
Ref	601660025	601660025
Lot	ETM06	ETM07
Mfg Date	2024-12	2024-12
Exp Date	2026-11	2026-11

*For the TrueLyse Buffer and TrueLyse MTB Ultima, the manufacturer reports that at the time of the study, the product had not been design locked and was under validation.

Table S3. STARD checklist

Section & Topic	No	Item	Reported section
TITLE OR ABSTRACT			
	1	Identification as a study of diagnostic accuracy using at least one measure of accuracy (such as sensitivity, specificity, predictive values, or AUC)	Title
ABSTRACT			
	2	Structured summary of study design, methods, results, and conclusions (for specific guidance, see STARD for Abstracts)	Abstract
INTRODUCTION			
	3	Scientific and clinical background, including the intended use and clinical role of the index test	Introduction
	4	Study objectives and hypotheses	Introduction
METHODS			
Study design	5	Whether data collection was planned before the index test and reference standard were performed (prospective study) or after (retrospective study)	Study design and participants
Participants	6	Eligibility criteria	Study design and participants
	7	On what basis potentially eligible participants were identified (such as symptoms, results from previous tests, inclusion in registry)	Study design and participants
	8	Where and when potentially eligible participants were identified (setting, location and dates)	Study design Results: participants
	9	Whether participants formed a consecutive, random or convenience series	Study design and participants
Test methods	10a	Index test, in sufficient detail to allow replication	Index test
	10b	Reference standard, in sufficient detail to allow replication	Reference standard and comparator tests/ Supplementary methods
	11	Rationale for choosing the reference standard (if alternatives exist)	Reference standard and comparator test
	12a	Definition of and rationale for test positivity cut-offs or result categories of the index test, distinguishing pre-specified from exploratory	Index tests
	12b	Definition of and rationale for test positivity cut-offs or result categories of the reference standard, distinguishing pre-specified from exploratory	Data analysis
	13a	Whether clinical information and reference standard results were available to the performers/readers of the index test	Index tests
	13b	Whether clinical information and index test results were available to the assessors of the reference standard	Reference standard and comparator tests
Analysis	14	Methods for estimating or comparing measures of diagnostic accuracy	Data analysis
	15	How indeterminate index test or reference standard results were handled	Data analysis; Figure 1
	16	How missing data on the index test and reference standard were handled	Data analysis; Figure 1

	17	Any analyses of variability in diagnostic accuracy, distinguishing pre-specified from exploratory	Data analysis
	18	Intended sample size and how it was determined	Data analysis
RESULTS			
Participants	19	Flow of participants, using a diagram	Figure 1
	20	Baseline demographic and clinical characteristics of participants	Table 1
	21a	Distribution of severity of disease in those with the target condition	Table 1
	21b	Distribution of alternative diagnoses in those without the target condition	N/A (did not evaluate)
	22	Time interval and any clinical interventions between index test and reference standard	Specimen Collection
Test results	23	Cross tabulation of the index test results (or their distribution) by the results of the reference standard	Tables 2, 4, S4, S7, S8
	24	Estimates of diagnostic accuracy and their precision (such as 95% confidence intervals)	Tables 2, 3, 4, S6
	25	Any adverse events from performing the index test or the reference standard	N/A
DISCUSSION			
	26	Study limitations, including sources of potential bias, statistical uncertainty, and generalisability	Discussion
	27	Implications for practice, including the intended use and clinical role of the index test	Conclusion
OTHER INFORMATION			
	28	Registration number and name of registry	Data analysis
	29	Where the full study protocol can be accessed	On request
	30	Sources of funding and other support; role of funders	Note – Financial support.

Table S4. Diagnostic accuracy of sputum swabs on the MiniDock MTB assay for detection of TB, as compared with the reference standard of TB culture, by sub-group

	**Sensitivity %<br>(95% CI, n/N)**	**Specificity %<br>(95% CI, n/N)**
Overall*	86% (79-91%)<br>114/132	97% (96-98%)<br>842/866
People living with HIV	88% (64-97%)<br>14/16	97% (93-99%)<br>167/172
People without HIV**	86% (79-91%)<br>94/109	98% (96-99%)<br>597/612
People enrolled at health facilities	87% (79-92%)<br>87/100	98% (97-99%)<br>561/571
People enrolled at ACF events	84% (68-93%)<br>27/32	97% (94-98%)<br>283/293
People reporting cough > 2 weeks	92% (85-96%)<br>95/103	97% (95-99%)<br>346/355
People not reporting cough > 2 weeks	66% (47-80%)<br>19/29	98% (96-99%)<br>498/509
People reporting any of W4SS	88% (81-92%)<br>112/128	98% (96-99%)<br>695/712
People not reporting any of W4SS	50% (15-85%)<br>2/4	98% (94-99%)<br>149/152

* 2 with invalid MiniDock MTB sputum swab results

** not including 101 people with HIV status unknown

W4SS: WHO-recommended four symptom screen (cough, fever, night sweats, weight loss)

Table S5. Diagnostic accuracy of tongue swabs on the MiniDock MTB assay for detection of TB, as compared with the reference standard of TB culture, by sub-group

	**Sensitivity % (95% CI, n/N)**	**Specificity % (95% CI, n/N)**
Overall*	76% (68-82%) 100/132	99% (98-99%) 856/865
People living with HIV**	56% (33-77%) 9/16	100% (98-100%) 173/173
People without HIV**	80% (71-86%) 87/109	99% (98-99%) 606/613
People enrolled at health facilities	80% (71-87%) 80/100	100% (99-100%) 572/573
People enrolled at ACF events	63% (45-77%) 20/32	97% (95-99%) 284/292
People with supervised self-collected swab	75% (66-82%) 84/112	99% (98-99%) 714/722
People with healthcare worker collected swab	80% (58-92%) 16/20	99% (96-100%) 142/143
People reporting cough > 2 weeks	83% (74-89%) 85/103	99% (98-100%) 351/354
People not reporting cough > 2 weeks	52% (34-69%) 15/29	99% (98-100%) 505/511
People reporting any of W4SS	77% (69-84%) 99/128	99% (98-99%) 705/713
People not reporting any of W4SS	25% (5-70%) 1/4	99% (96-100%) 151/152

* 1 with invalid MiniDock MTB tongue swab result

** not including 101 people with HIV status unknown

W4SS: WHO-recommended four symptom screen (cough, fever, night sweats, weight loss); ACF: Active case finding

Table S6. Diagnostic accuracy of sputum swabs on the MTB Ultima assay for detection of TB, as compared with the reference standard of TB culture, by sub-group

	**Sensitivity %** **(95% CI, n/N)**	**Specificity %** **(95% CI, n/N)**
Overall	84% (76-89%) 97/116	97% (96-98%) 633/650
People living with HIV	79% (52-92%) 11/14	99% (96-100%) 157/159
People without HIV**	85% (76-90%) 83/98	97% (95-98%) 449/462

** not including people with HIV status unknown

Table S7. Diagnostic accuracy of tongue swabs on the MTB Ultima assay for detection of TB, as compared with the reference standard of TB culture, by sub-group

	**Sensitivity %** **(95% CI, n/N)**	**Specificity %** **(95% CI, n/N)**
Overall	74% (64-82%) 67/91	98% (96-98%) 467/476
People living with HIV**	55% (28-79%) 6/11	98% (98-100%) 129/131
People without HIV**	76% (65-84%) 60/79	98% (98-99%) 326/333
People with supervised self-collected swab	73% (63-82%) 60/82	98% (98-99%) 399/407
People with healthcare worker collected swab	78% (45-94%) 7/9	99% (96-100%) 68/69

** not including people with HIV status unknown

Table S8. Diagnostic agreement of sputum and tongue swabs on the MTB Ultima assay for detection of TB, as compared with sputum on the Xpert MTB/RIF Ultra assay, by result grade

	Positive percent agreement			Negative percent agreement		
	% (95% CI)		n/N	% (95% CI)		n/N
**Sputum swabs** (n=814)						
MTB Detected						
High	96%	(88-99%)	55/57			
Medium	90%	(71-97%)	19/21			
Low	85%	(68-94%)	23/27			
Very low	67%	(35-89%)	6/9			
Trace	22%	(9-45%)	4/18			
MTB Not detected				98.4%	(97-99%)	673/684
**Tongue swabs** (n=605)						
MTB Detected						
High	90%	(78-96%)	44/49			
Medium	81%	(57-93%)	13/16			
Low	52%	(32-72%)	11/21			
Very low	25%	(5-70%)	1/4			
Trace	0%	(0-30%)	0/9			
MTB Not detected				98.6%	(97-99%)	499/506

Table S9. Test success rate, MiniDock MTB, by swab type

	Overall n/N (%)
**MiniDock MTB sputum swab**	
Tongue swab initial invalid result	11/1093 (1.0%)
Repeat sputum swab result as invalid	2/11 (18.2%)
**MiniDock MTB tongue swab**	
Tongue swab initial invalid result	10/1096 (0.9%)
Repeat sputum swab result as invalid	1/10 (10.0%)

Table S10. Time to result of MTB positive sputum and tongue swabs on the MiniDock MTB instrument, overall and by Ultra sputum semi-quantitative grade

	**Sputum swab**			**Tongue swab**		
	Number	Average time to results, minutes	SD, minutes	Number	Average time to results, minutes	SD, minutes
**Overall***	129	14.6	5.1	99	15.1	4.7
Ultra MTB detected, grade						
High	55	11.7	0.9	53	14.0	3.7
Medium	19	11.9	0.6	17	14.1	3.1
Low	28	14.2	4.1	22	16.6	5.4
Very Low	5	22.9	4.7	2	21.3	5.3
Trace	6	21.4	5.7	0		
Ultra MTB Not Detected	16	23.4	3.6	5	22.3	6.0

*11 positive sputum swab results with no time recorded; 10 positive tongue swab results with no time recorded
Notes: All swabs with MiniDock MTB negative results had a test time of 25 minutes (not included in this table). The Thermolyse step prior to MiniDock testing is 5 minutes for all specimens.

Table S11. Ambient temperature measured at the time of swab testing with the MiniDock MTB device, by setting and test result

	Total			Active Case Finding			Health Facilities		
	N	Average (◦C)	Range (◦C)	N	Average (◦C)	Range (◦C)	N	Average (◦C)	Range (◦C)
**Sputum swab**									
**Overall**	**982**	**29.0**	**(13 - 48.4)**	**377**	**35.4**	**(23 - 48.4)**	**605**	**24.6**	**(13 - 35)**
By result									
Negative	878	29.1	(13 - 48.4)	347	35.4	(23 - 48.4)	531	24.6	(13 - 35)
Positive	99	27.9	(17 - 43.7)	27	34.8	(23 - 43.7)	72	24.9	(17 - 32)
Invalid	5	33.3	(26.2 - 40.4)	3	37.9	(36.3 - 40.4)	2	26.5	(26.2 - 26.8)
**Tongue swab**									
**Overall**	**992**	**29.5**	**(17 - 44.4)**	**377**	**36.5**	**(21.7 - 44.4)**	**615**	**24.7**	**(17 - 35)**
By result									
Negative	860	29.6	(17 - 44.4)	336	36.7	(21.7 - 44.4)	524	24.7	(17 - 35)
Positive	129	28.4	(17 - 44.4)	41	35.3	(24.7 - 44.4)	88	24.6	(17 - 30)
Invalid	3	23.3	(17 - 27)	0			3	23.3	(17 - 27)
